# Sin-QuEChERS Nano净化柱结合气相色谱-串联质谱法快速筛查石斛中84种农药残留

**DOI:** 10.3724/SP.J.1123.2021.12010

**Published:** 2022-06-08

**Authors:** Quan ZHANG, Shan BI, Yutian WU, Lei LI, Yibing ZHOU, Liya LIU, Wenzheng LIU, Qingyuan CHEN, Xue ZHOU, Hua GUO

**Affiliations:** 1.贵州省疾病预防控制中心实验中心, 贵州 贵阳 550004; 1. Experimental Center, Guizhou Center for Disease Control and Prevention, Guiyang 550004, China; 2.贵州医科大学药学院, 贵州 贵阳 550025; 2. School of Pharmacy, Guizhou Medical University, Guiyang 550025, China

**Keywords:** 气相色谱-串联质谱, Sin-QuEChERS Nano净化柱, 快速筛查, 农药残留, 石斛, gas chromatography-tandem mass spectrometry (GC-MS/MS), Sin-QuEChERS Nano purification column, rapid screening, pesticide residues, dendrobium

## Abstract

利用Sin-QuEChERS Nano净化柱结合气相色谱-串联质谱(GC-MS/MS)分析,建立了石斛基质中84种不同极性农药残留的快速筛查方法。比较了采用不同的提取溶剂(1%乙酸乙腈、丙酮)和不同的提取方式(加水浸泡和不加水浸泡)下目标物的提取效率。利用金钗石斛样品系统比较了Sin-QuEChERS Nano法与经典的基质固相分散法(dSPE)、固相萃取法(SPE)、QuEChERS法的净化效果及提取回收率,以及净化效果较好的Sin-QuEChERS Nano法与dSPE法基质效应的差异。目标物经DB-1701MS石英毛细管色谱柱(30 m×0.25 mm×0.25 μm)程序升温分离,GC-MS/MS多反应监测(MRM)模式检测,基质匹配溶液外标法定量。通过GC-MS/MS检测方法对金钗石斛和铁皮石斛中的84种代表性农药进行了方法学验证。结果表明:各目标物在不同范围内呈良好的线性相关,相关系数(*r*^2^)均>0. 990,方法的检出限(LOD, *S/N*=3)为1.5~5.8 μg/kg,方法的定量限(LOQ, *S/N*=10)为5.0~15.0 μg/kg。在两个水平下,目标农药的加标回收率为68.7%~116.2%,相对标准偏差(RSD, *n*=6)均低于15%。与其他经典的前处理方法相比,Sin-QuEChERS Nano法在净化效果方面表现更好,该法不仅可以有效去除色素、有机酸、碱性干扰物等物质,还可以节省样品制备时间,避免溶剂转移造成的损失,无需进一步涡旋或离心,是一种简单而有效的提取物纯化程序。该方法灵敏、快速、简便、可靠,有效地提高了石斛中农药快速筛查时的检测效率,具有较强的实际应用价值。此外,所开发的方法可以进一步扩展目标农药的类型,并可以用于检测其他更多食品及中药材中的农药残留。

## Abstract (en)

A rapid screening method for 84 pesticide residues in dendrobium perfringens parent material with different polarities was developed using a Sin-QuEChERS Nano clean-up column combined with gas chromatography-tandem mass spectrometry (GC-MS/MS). The differences in extraction efficiency of the targets were compared with different extraction solvents (acetonitrile containing 1% acetic acid, acetone) and methods (immersion with or without water). The purification effect and extraction recoveries of Sin-QuEChERS Nano method and classical dispersive solid-phase extraction (dSPE), solid-phase extraction (SPE) and QuEChERS were systematically compared using *Dendrobium nobile* samples. The differences in matrix effects between the Sin-QuEChERS Nano method, which was more effective in purification, and the dSPE method were also analyzed. The purification effects of three commercially available Sin-QuEChERS Nano purification columns (simple matrix purification column, complex matrix purification column and herbal purification column) were compared. The applicability of the purification methods were also verified by using different parts of *Dendrobium nobile* samples (stems, leaves and flowers). From the results, it could be concluded that weighing 2.00 g and the samples in 5 mL of water for 20 min, followed by extraction with acetonitrile containing 1% acetic acid was more effective. The average extraction recovery of the target components by Sin-QuEChERS Nano purification method was 90.5%, which further identified Sin-QuEChERS Nano-Chinese medicine purification column as the preferred purification column for dendrobium purification. The target components were separated by a DB-1701MS quartz capillary column (30 m×0.25 mm×0.25 μm) with programmed temperature rise, detected by multiple reaction monitoring (MRM) mode, and quantified by matrix-matched solution external standard method. The GC-MS/MS assay was used for the methodological validation of the 84 representative pesticides within *Dendrobium officinale* and *Dendrobium nobile* was carried out by GC-MS/MS detection method. The results indicated that the targets showed excellent linear correlation in different scopes with correlation coefficients (*r*^2^) >0. 990. The limits of detection (LODs, *S/N*=3) of the method were 1.5 to 5.8 μg/kg, and the limits of quantification (LOQs, *S/N*=10) ranged from 5.0 to 15.0 μg/kg. The spiked recoveries of the target pesticides under different spiked levels were 68.7%-116.2%, and the relative standard deviations (RSDs, *n*=6) were less than 15%. Compared to other typical pretreatment methods, the Sin-QuEChERS Nano method provided better performance in terms of purification. The method not only effectively removed pigments, organic acids, and alkaline interferents, but also saved preparation time. Losses due to solvent transfer were also avoided and no further vortexing or centrifugation was required, making it a simplified and effective extraction and purification procedure. The method was sensitive, rapid, simple and reliable. It effectively improved the detection efficiency during the rapid screening of pesticides in dendrobium and presented a strong practical application value. In addition, the developed method could further expand the types of target pesticides and could be used to detect more pesticide residues in foods and Chinese herbal medicine. The established Sin-QuEChERS Nano method was used for the analysis of authentic samples. The applicability of the method was evaluated by analyzing a total of 80 samples collected from Anlong, Libo, Dushan, and Yanhe County in Guizhou Province. The types of samples included dendrobium maple, *Dendrobium nobile* (flowers, stems, leaves) and *Dendrobium officinale* (flowers, stems, leaves, powder, tablets). At least one pesticide residue was detected in 12 samples, with a detection rate of 15%. The five pesticides with higher detection rates and residues were chlorpyrifos (0.08-0.5 mg/kg), chlorothalonil (0.06-3.2 mg/kg), propanil zinc (0.03-0.15 mg/kg), methyl parathion (0.04-0.23 mg/kg) and cyhalothrin (0.10-2.68 mg/kg). Except for the pesticides in maximum residue limits (MRLs), the pesticide residues detected from dendrobium samples were below the limits set by Chinese national standard (GB 2763-2021) and local standard DBS 52/048-2020.

兰科(*Orchidaceae*)石斛属(*Dendrobium Sw*.)植物为多年生草本^[1]^。石斛属在全世界大约有150种,划分为40个组,主要分布于亚洲及大洋洲的热带和亚热带区域,部分种类也分布于亚高山地区^[2]^。在中国石斛种属有78种,主要分布于华南、华东及西南等地^[3]^。贵州省有25种属,常见的主要以铁皮石斛、金钗石斛为主,其中铁皮石斛被誉为“九大仙草之首”,为历年版《中华人民共和国药典》收载的珍稀名贵中药材^[4]^。现代药理研究表明,石斛中含有多糖、生物碱、氨基酸等多种活性成分,具有抗氧化、抗肿瘤、增强免疫等诸多作用^[5⇓⇓-8]^。近些年石斛不仅作为传统中药使用,更作为原料广泛用于食品、保健品等行业^[9,10]^。由于过度采挖,野生石斛资源已濒临枯竭,目前主要以大面积人工仿野生栽培来满足市场需求,但在种植过程中常用农药来防治各种害虫,而过量使用农药不仅会造成环境污染,还会残留在药材内,危害人类健康^[11]^。因此,为保障石斛原材料的用药安全,建立多种农药残留快速筛查的方法就显得非常重要。

农药残留分析是一项复杂的痕量分析技术,随着检测样品基质的复杂化现象越来越突出,传统的提取净化技术已远远满足不了现代农药残留分析的要求^[12,13]^,如何应对日益复杂的样品基质前处理及其痕量分析已成为业内一大挑战^[14]^。通常样品前处理是农药残留检测的关键,占整个分析过程2/3的时间^[15]^。方法主要有固相萃取法(SPE)^[16]^、凝胶渗透色谱法(GPC)^[17]^、QuEChERS^[18]^、分散固相萃取法(dSPE)^[19]^等,其中QuEChERS方法问世后目前已逐步成为多组分农药残留测定最流行的前处理方法^[20]^。原理是利用吸附剂填料与基质中的杂质相互作用,吸附杂质,从而达到除杂净化的目的。常用的净化剂填料主要有*N*-丙基乙二胺(PSA)^[21]^、十八烷基硅烷键合硅胶(C_18_)^[22]^及石墨化炭黑(GCB)^[23]^等。而Sin-QuEChERS Nano疏水型净化柱、MPFC-QuEChERS超滤型净化柱^[24]^是通过QuEChERS法进行技术改良后的产品,它是将一种具备强度高、韧性大及比表面积大等多种优点的新型多壁碳纳米管净化填料(multi-walled carbon nanotubes, MWCNTs)与其他净化填料结合^[25]^,巧妙地将底部为漏斗状的柱体与实验室常用的50 mL离心管组成密封体系,将传统QuEChERS方法中萃取、净化两步“化简唯一”,从而避免因溶剂转移所带来的结果损失,真正实现一步净化。

目前Sin-QuEChERS Nano法在蔬菜及水果基质中有少数的应用^[26,27]^,但是在石斛等复杂基质中的应用还未见报道。所以本研究以石斛基质为代表,通过比较本实验室所建立的几种经典样品前处理方法(SPE、dSPE、QuEChERS)的净化效果及回收率差异,突出Sin-QuEChERS Nano法在石斛基质净化过程中的优势性。以目前实际用于石斛种植中的农药种类,选择涵盖有机氯类、有机磷类、菊酯类、氨基甲酸酯类等84种代表性农药作为目标物,通过对质谱、色谱条件、基质效应、提取方式及净化方法的考察与优化,建立了Sin-QuEChERS Nano结合GC-MS/MS对石斛中多种农药残留的快速筛查方法,对不同产地、不同部位的80份金钗石斛和铁皮石斛开展监测,为后续中国国家标准修订石斛中农药最大残留限量提供参考。

## 1 实验部分

### 1.1 仪器与试剂

Scion-TQ三重四极杆质谱联用仪(美国BRUKER公司); FW100型高速万能粉碎机(天津市泰斯特仪器有限公司); GL-22MS型高速冷冻离心机(上海卢湘仪离心机仪器有限公司)。

33种药典中农药定制标准溶液(溶液丙酮,天津阿尔塔科技有限公司); 10种氨基甲酸酯标准溶液(溶液丙酮,农业部环境保护科研监测所); 16种有机磷标准溶液(溶液丙酮,农业部环境保护科研监测所); 7种菊酯标准溶液(溶液正己烷,100 μg/mL,美国O2si公司),其余26种农药标准品均购自农业部环境保护科研监测所(溶液丙酮,100 μg/mL);乙腈、正己烷、丙酮(色谱纯,美国TEDIA公司); CNW dSPE萃取包及纯化管(萃取包内填料为6 g硫酸镁及1.5 g醋酸钠,纯化管内含150 mg MgSO_4_、50 mg PSA、50 mg GCB、50 mg C_18_)、缓冲盐萃取包(含4 g MgSO_4_、1 g NaCl、0.5 g柠檬酸氢二钠、1.5 g柠檬酸钠)(上海安谱实验科技股份有限公司); Cleanert PC/NH_2_固相萃取柱(1000 mg/6 mL,天津博纳艾杰尔公司); LUMTECH M-PFC复杂基质超滤柱(3 mL)、LUMTECH Sin-QuEChERS Nano-Herb中药农残净化柱(内含MWCNTs、PSA及GCB填料)(北京绿绵科技有限公司)。金钗石斛、铁皮石斛样品采集于中国贵州省安龙县、荔波县、独山县、沿河县。

### 1.2 标准储备液的配制

分别用1 mL胖度移液管吸取上述4种混合标准溶液及26种单标准溶液各1 mL后混匀,合并后的农药混合标准储备液总体积为30 mL,于-4 ℃冷藏保存备用,其中84种目标组分中甲萘威、灭多威及甲胺磷等8个组分在混合标准储备液中重复,所以在后续计算时已把浓度做加和处理。临用前使用倍比稀释法经1.3节中Sin-QuEChERS Nano法处理的空白提取液稀释成基质匹配混合标准工作溶液。

### 1.3 样品前处理过程

选取500 g以上代表性样品,利用高速粉碎机粉碎后置于自封袋中,标注样品编号,备用。

dSPE法:准确称取2.00 g试样于50 mL离心管中,加入10 mL 1%乙酸乙腈涡旋振荡2 min,超声提取15 min,然后加入萃取包(填料为6.0 g硫酸镁及1.5 g乙酸钠),涡旋振荡1 min,以10000 r/min离心5 min,准确吸取上清液1 mL加入到纯化管中(150 mg MgSO_4_、50 mg PSA、50 mg GCB、50 mg C_18_),然后加入30 μg/mL环氧七氯内标40 μL,涡旋振荡1 min,以10000 r/min离心5 min,上清液过0.22 μm滤膜后待GC-MS/MS分析。

M-PFC法:准确称取2.00 g试样于5 mL离心管中,加入3 mL乙腈,超声提取10 min,以10000 r/min离心5 min。移取上述提取液2 mL置于5 mL离心管中,向上缓慢抽动M-PFC柱的注射杆,使2 mL提取液全部经过填料层,往复过柱两次,M-PFC柱注射器端连接0.22 μm滤膜,置于进样小瓶上方,然后缓慢推动注射杆,即得待测液,待GC-MS/MS分析。

Sin-QuEChERS Nano法:准确称取2.00 g试样,加入5 mL水混匀后浸泡20 min,再加入10 mL 1%乙酸乙腈和缓冲盐萃取包,超声提取10 min,以10000 r/min离心5 min,将Sin-QuEChERS Nano柱插入50 mL离心管内,缓慢按压至刻度处(储液管内净化液约4 mL),用注射器吸取上清液过0.22 μm滤膜,待GC-MS/MS分析。具体Sin-QuEChERS Nano结构示意图如图1所示。

**图1:**
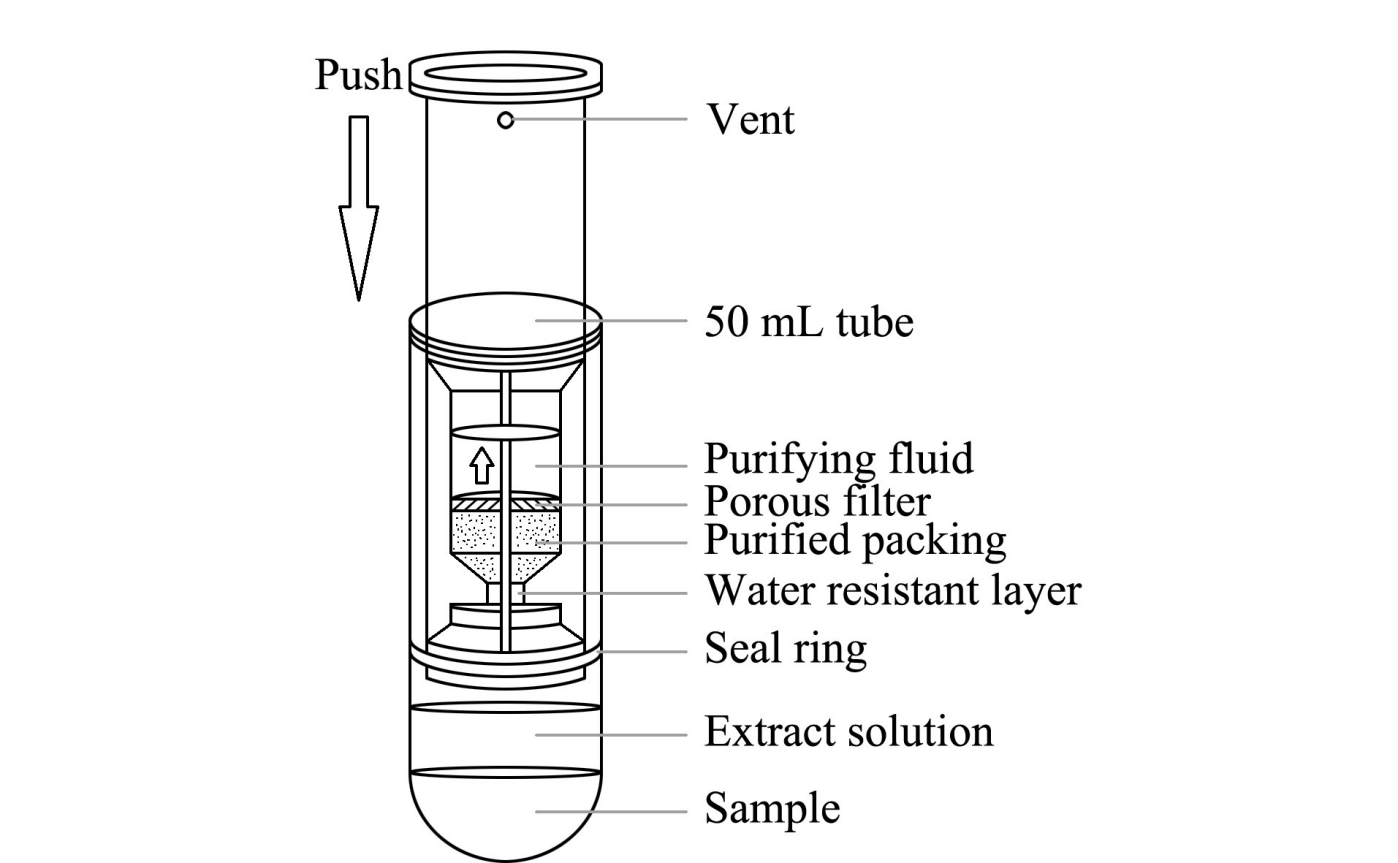
Sin-QuEChERS Nano净化柱的示意图

SPE法:准确称取2.00 g试样,加入5 mL水混匀后浸泡20 min,再加入10 mL乙腈和3 g氯化钠,超声提取10 min,振荡0.5 min,待液液分层后加入4 g无水硫酸镁,迅速旋上瓶盖,涡旋振荡1 min,以10000 r/min离心5 min,取上清液5 mL,浓缩至1 mL。先用5 mL淋洗液活化PC/NH_2_柱,然后用3 mL淋洗液溶解平底烧瓶中的蒸发残留物,合并洗涤液并转移到PC/NH_2_柱上,当液面降低到固相萃取柱筛板表面后加入淋洗液10 mL,收集淋洗液于15 mL离心管中,浓缩液用氮气吹至近干后用加入1.0 mL丙酮,过0.22 μm滤膜,待GC-MS/MS分析。

### 1.4 仪器条件

#### 1.4.1 色谱条件

色谱柱:Agilent DB-1701MS石英毛细管色谱柱(30 m×0.25 mm×0.25 μm),载气:高纯氦气,纯度≥99.999%;碰撞气(CID):氩气,分流模式:不分流进样,进样口温度:250 ℃,程序升温条件:起始温度60 ℃,保持1 min,以40 ℃/min升至120 ℃,再以5 ℃/min升至280 ℃,保持8.5 min,共43 min。流速:1 mL/min;进样量:1.0 μL。

#### 1.4.2 质谱条件

电离模式:电子轰击电离(EI);电离能量:70 eV;离子监测模式:多反应监测(MRM);离子源温度:220 ℃;传输线温度:280 ℃;溶剂延迟:5.0 min。目标物的质谱参数见表1。石斛加标样品的总离子流图见图2。

**表1:** 84种农药的保留时间及质谱参数

No.		Pesticide	Chemical formula		*M*_r_		*t*_R_/min		CAS No.		Ion pairs (*m/z*)				CEs/eV
1		methomyl	C_5_H_10_N_2_O_2_S		162.21		5.42		16752-77-5		105>		58^*^, 105>88		15, 15
2		metolcarb	C_9_H_11_NO_2_		165.19		6.02		1129-41-5		108>		80^*^, 108>90		10, 15
3		entrofolan	C_11_H_15_NO_2_		193.24		7.42, 14.52		2631-40-5		121>		77^*^, 136>103		20, 25
4		metrifonate	C_4_H_8_Cl_3_O_4_P		257.44		7.89		52-68-6		185>		93^*^, 185>63		10, 15
5		dichlorvos	C_4_H_7_Cl_2_O_4_P		220.98		7.93		62-73-7		109>		79^*^, 185>93		5, 10
6		carbofuran	C_12_H_15_NO_3_		221.25		8.42, 19.00		1563-66-2		164>		149^*^, 149>121		10, 5
7		biphenyl	C_12_H_10_		154.21		9.51		92-52-4		154>		153^*^, 154>128		10, 25
8		methamidophos	C_2_H_8_NO_2_PS		141.13		10.75		10265-92-6		141>		95^*^, 141>64		10, 20
9		demeton	C_8_H_19_O_3_PS_2_		258.34		14.75		126-75-0		88>		60^*^, 170>114		10, 10
10		ethoprophos	C_8_H_19_O_2_PS_2_		242.34		15.31		13194-48-4		158>		97^*^, 158>81		18, 15
11		carbaryl	C_12_H_11_NO_2_		201.22		15.52, 23.34		63-25-2		144>		115^*^, 144>116		20, 10
12		fenobucarb	C_12_H_17_NO_2_		207.27		15.84		3766-81-2		121>		77^*^, 150>103		20, 25
13		chlordimeform	C_10_H_13_ClN_2_		196.68		15.86		6164-98-3		152>		117^*^, 196>181		15, 5
14		phorate	C_7_H_17_O_2_PS_3_		260.38		16.41		298-02-2		121>		93^*^, 260>175		5, 20
15		propoxur	C_11_H_15_NO_3_		209.24		16.45		114-26-1		110>		64^*^, 152>109		15, 10
16		sulfotep	C_8_H_20_O_5_P_2_S_2_		322.32		16.71		3689-24-5		322>		266^*^, 322>146		10, 10
17		*α*-hexachlorocyclohexane	C_6_H_6_Cl_6_		290.83		17.09		319-84-6		217>		181^*^, 219>147		10, 20
18		bendiocarb	C_11_H_13_NO_4_		223.23		17.34		22781-23-3		166>		151^*^, 126>52		10, 15
19		quintozine	C_6_Cl_5_NO_2_		295.34		17.59		82-68-8		249>		214^*^, 237>143		15, 20
20		terbufos	C_9_H_21_O_2_PS_3_		288.43		17.87		13071-79-9		231>		129^*^, 231>97		30, 30
21		dithianon	C_12_H_21_N_2_O_3_PS		304.35		18.15		333-41-5		304>		179^*^, 304>137		10, 35
22		lindane	C_6_H_6_Cl_6_		290.83		18.71		58-89-9		217>		181^*^, 219>147		10, 20
23		omethoate	C_5_H_12_NO_4_PS		213.19		18.91		1113-02-6		156>		110^*^, 156>79		10, 25
24		atrazine	C_8_H_14_ClN_5_		215.68		19.01		1912-24-9		215>		200^*^, 215>172		10, 15
25		cyromazine	C6H_10_N_6_		166.18		19.59		66215-27-8		151>		109^*^, 151>109		15, 15
26		pirimicarb	C_11_H_18_N_4_O_2_		238.29		20.13		23103-98-2		238>		166^*^, 166>123		10, 10
27		isazofos	C_9_H_17_ClN_3_O_3_PS		313.74		20.16		42509-80-8		161>		146^*^, 257>162		10, 20
28		aldrin	C_12_H_8_Cl_6_		364.91		20.32		309-00-2		255>		220^*^, 263>193		20, 30
29		chlorpyrifos-methyl	C_7_H_7_Cl_3_NO_3_PS		322.53		20.55		5598-13-0		286>		93^*^, 286>208		20, 10
30		dimethoate	C_5_H_12_NO_3_PS_2_		229.26		20.61		60-51-5		125>		125^*^, 125>79		5, 10
31		monocrotophos	C_7_H_14_NO_5_P		223.16		20.62		6923-22-4		127>		79^*^, 127>95		18, 18
32		fenchlorphos	C_8_H_8_C_l3_O_3_PS		321.55		20.82		299-84-3		285>		270^*^, 287>272		15, 15
No.		Pesticide	Chemical formula		*M*_r_		*t*_R_/min		CAS No.		Ion pairs (*m/z*)				CEs/eV
33		indoxacarb	C_22_H_17_ClF_3_N_3_O_7_		527.83		21.45		144171-61-9		264>		232^*^, 264>148		5, 25
34		pirimiphos-methyl	C_11_H_20_N_3_O_3_PS		305.33		21.46		29232-93-7		290>		233^*^, 290>125		10, 35
35		chlorothalonil	C_8_Cl_4_N_2_		265.91		21.57		1897-45-6		266>		231^*^, 266>133		18, 30
36		*β*-hexachlorocyclohexane	C_6_H_6_C_6_		290.83		22.04		319-85-7		181>		145^*^, 219>183		15, 10
37		clorpyrifos	C_9_H_11_Cl_3_NO_3_PS		350.59		22.08		2921-88-2		197>		169^*^, 314>166		15, 35
38		parathion-methyl	C_8_H_10_NO_5_PS		263.21		22.33		298-00-0		263>		109^*^, 263>246		15, 5
39		fenthion	C_10_H_15_O_3_PS_2_		278.33		22.36		55-38-9		278>		109^*^, 278>125		20, 18
40		dicofol	C_14_H_9_Cl_5_O		370.49		22.63		115-32-2		139>		111^*^, 251>139		15, 10
41		*δ*-hexachlorocyclohexane	C_6_H_6_Cl_6_		290.83		22.71		319-86-8		181>		145^*^, 219>183		15, 10
42		malaoxon	C_10_H_19_O_6_PS_2_		330.36		22.97		121-75-5		126.9>		99^*^, 173>99		10, 18
43		fenitrothion	C_9_H_12_NO_5_PS		277.23		23.18		122-14-5		260>		125^*^, 277>109		15, 20
44		parathion	C_10_H_14_NO_5_PS		291.26		23.83		56-38-2		138.9>		109^*^, 291>109		5, 15
45		*α*-endosulfan	C_9_H_6_Cl_6_O_3_S		406.93		24.02		959-98-8		241>		206^*^, 241>170		15, 25
46		isofenphos-methyl	C_14_H_22_NO_4_PS		331.37		24.04		99675-03-3		241>		199^*^, 241>93		10, 50
47		phorat-sulfoxide	C_7_H_17_O_3_PS_3_		276.38		24.09		2588/3/6		199>		143^*^, 97>65		10, 20
48		quintiofos	C_17_H_16_NO_2_PS		329.35		24.38		1776-83-6		157>		129^*^, 157>102		15, 15
49		isocarbophos	C_11_H_16_NO_4_PS		289.29		24.53		24353-61-5		135.9>		108^*^, 230>230		15, 5
50		*p*,*p'*-dichlorodiphenyldichloroethane	C_14_H_8_Cl_4_		318.03		24.98		72-55-9		316>		246^*^, 246>211		20, 20
51		phorate-sulfone	C_7_H_17_O_4_PS_3_		292.38		24.98		2588-04-7		125>		97^*^, 153>97		5, 10
52		dieldrin	C_12_H_8_Cl_6_O		380.91		25.39		60-57-1		277>		241^*^, 277>170		10, 40
53		profenofos	C_11_H_15_BrClO_3_PS		373.63		25.93		41198-08-7		139>		97^*^, 339>251		10, 25
54		procymidone	C_13_H_11_Cl_2_NO_2_		284.14		25.94		32809-16-8		96>		67^*^, 283>96		10, 10
55		methidathion	C_6_H_11_N_2_O_4_PS_3_		302.33		25.94		950-37-8		145>		85^*^, 145>58		10, 15
56		endrin-ketone	C_12_H_9_C_l5_O		346.46		26.14		72-20-8		263>		191^*^, 281>246		30, 15
57		phosfolan-methyl	C_5_H_10_NO_3_PS_2_		227.24		26.41		5120-23-0		227>		92^*^, 227>60		10, 30
58		*o*,*p'*-dichlorodiphenyltrichloroethane	C_14_H_9_Cl_5_		354.49		26.57		789-02-6		235>		165^*^, 235>199		15, 15
59		fipronil-desulfinyl	C_12_H_4_Cl_2_F_6_N_4_		389.08		26.89		205650-65-3		388>		333^*^, 333>281		15, 15
60		fenamiphos	C_13_H_22_NO_3_PS		303.36		26.92		22224-92-6		303>		154^*^, 303>139		18, 30
61		nitrofen	C_12_H_7_Cl_2_NO_3_		284.1		27.64		1836-75-5		285>		255^*^, 285>204		10, 15
62		*p*,*p'*-dichlorodiphenyldichloroethane	C_14_H_10_Cl_4_		320.04		27.91		72-52-4		235>		165^*^, 235>199		15, 15
63		*β*-endosulfan	C_9_H_6_Cl_6_O_3_S		406.93		28.01		33213-65-9		195>		159^*^, 241>206		10, 15
64		ethion	C_9_H_22_O_4_P_2_S_4_		384.48		28.19		563-12-2		231>		129^*^, 153>97		25, 10
65		*p*,*p'*-dichlorodiphenyltrichloroethane	C_14_H_9_Cl_5_		354.49		28.41		50-29-3		235>		165^*^, 235>200		15, 10
66		*τ*-fluvalinate	C_26_H_22_ClF_3_N_2_O_3_		502.91		28.63		102851-06-9		250>		250^*^, 250>200		5, 18
67		chlorfenapyr	C_15_H_11_BrClF_3_N_2_O		407.61		28.79		12453-73-0		247>		247^*^, 247>200		5, 25
68		fipronil-sulfide	C_12_H_4_Cl_2_F_6_N_4_S		421.15		29.21		120067-83-6		351>		255^*^, 351>228		20, 35
69		fipronil	C_12_H_4_Cl_2_F_6_N_4_OS		437.15		29.59		120068-37-3		367>		213^*^, 367>255		30, 15
70		bifenthrin	C_23_H_22_ClF_3_O_2_		422.87		29.85		82657-04-3		181>		166^*^, 181>115		10, 40
71		triazophos	C_12_H_16_N_3_O_3_PS		313.31		29.93		24017-47-8		161>		134^*^, 257>134		10, 20
72		thiodan-Sulfate	C_9_H_6_Cl_6_O_4_S		422.92		30.75		1031-07-8		272>		237^*^, 272>141		15, 35
73		fenpropathrin	C_22_H_23_NO_3_		349.42		31.26		39515-41-8		265>		210^*^, 265>181		10, 20
74		phosphonothioic acid	C_14_H_14_NO_4_PS		323.3		31.91		2104-64-5		169>		141^*^, 157>77		5, 20
75		phosmet	C_11_H_12_NO_4_PS_2_		317.32		32.39		732-11-6		160>		133^*^, 160>105		10, 18
76		cyhalothrin	C_23_H_19_ClF_3_NO_3_		449.85		33.39		91465-08-6		181>		152^*^, 181>127		20, 30
77		permethrin	C_21_H_20_Cl_2_O_3_		391.29		33.64		52645-53-1		183>		168^*^, 183>152		10, 20
78		phosalone	C_12_H_15_ClNO_4_PS_2_		367.81		33.65		2310-17-0		367>		182^*^, 367>138		10, 30
79		fipronil-sulfone	C_12_H_4_Cl_2_F_6_N_4_O_2_S		453.15		33.98		120068-36-2		383>		255^*^, 383>213		20, 32
80		cyfluthrin	C_22_H_18_Cl_2_FNO_3_		434.29		36.84		66359-37-5		226>		206^*^, 206>151		20, 15
81		cypermethrin	C_22_H_19_Cl_2_NO_3_		416.3		36.91		52315-07-8		181>		152^*^, 163>127		20, 5
82		coumaphos	C_14_H_16_ClO_5_PS		362.77		37.19		56-72-4		362>		109^*^, 362>226		15, 15
83		fenvalerate	C_25_H_22_ClNO_3_		419.9		39.55		51630-58-1		225>		147^*^, 225>119		10, 18
84		deltamethrin	C_22_H_19_Br_2_NO_3_		505.2		41.23		52918-63-5		172>		93^*^, 253>172		10, 10

CE: collison energy; * quantitative ion.

**图2:**
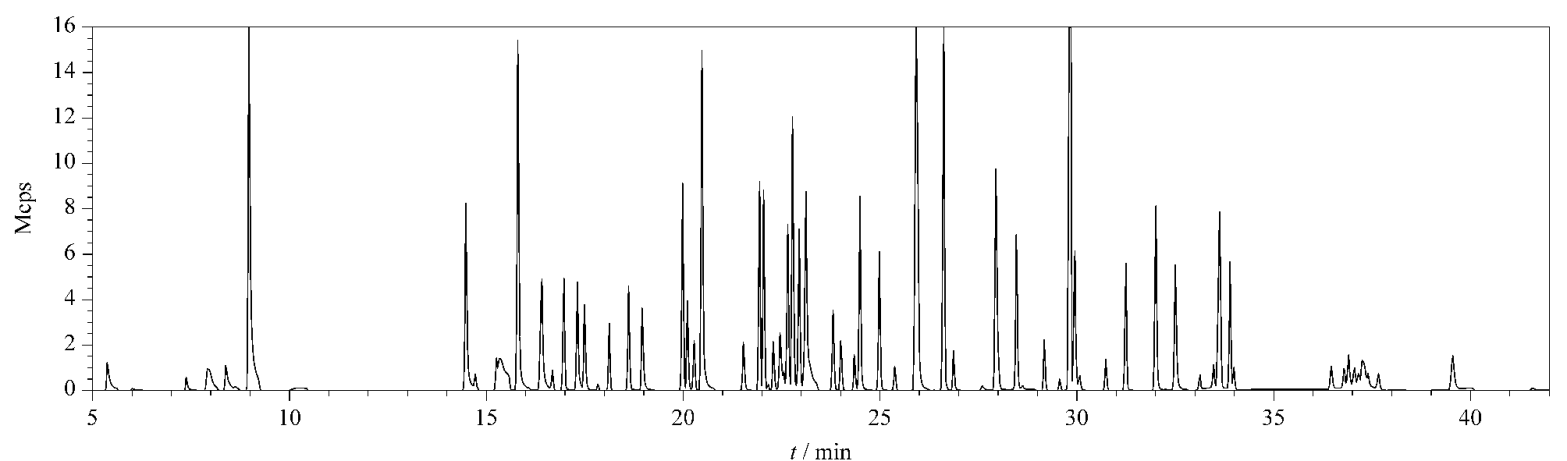
石斛加标样品的总离子流图

## 2 结果与讨论

### 2.1 净化方式的比较与优化

石斛类基质中含有色素、糖类、生物碱和有机酸等物质,这些干扰物在提取过程中会和目标物一起浸提出^[28]^。如果不进行后续的净化处理,不仅会对目标物的定性定量结果造成干扰,而且还会减少衬管的使用寿命和加重离子源的污染程度^[29]^。目前比较常用的净化方法主要有SPE法、QuEChERS法、dSPE法等。

首先将石斛样品在全扫描(Full Scan)模式下进样分析比较,4种净化方式的净化效果,具体如图3所示;将石斛加标样品在MRM模式下进样分析,比较4种净化方式的平均回收率差异。

**图3:**
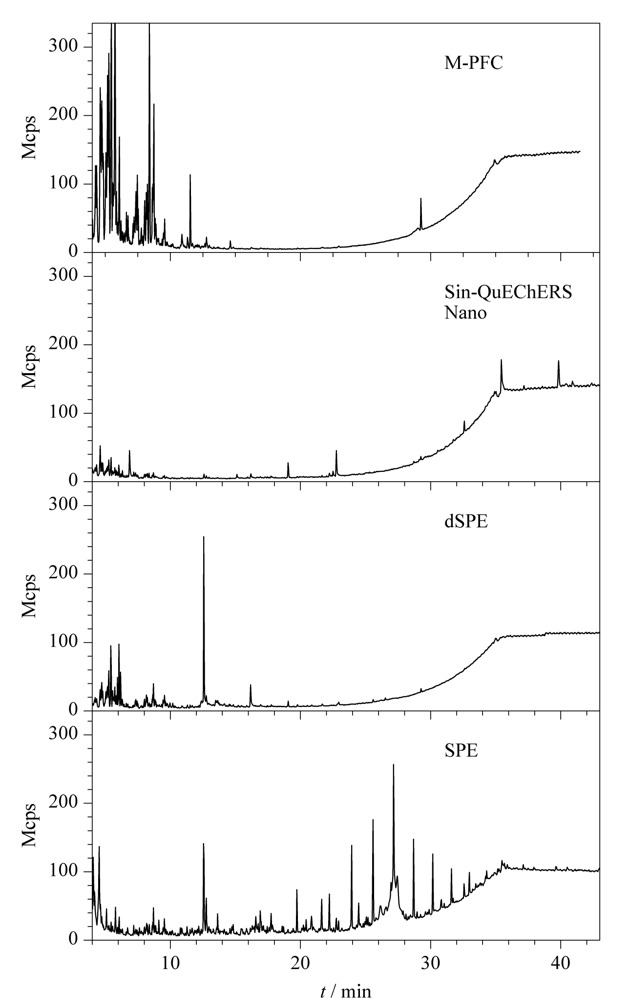
经不同净化方式处理后石斛提取液在全扫描模式下的总离子流色谱图

Sin-QuEChERS Nano法、M-PFC法、dSPE法及SPE法的平均回收率分别为90.5%、82.8%、71.2%和65.3%。从图3可以看出,石斛基质经Sin-QuEChERS Nano法及dSPE法净化后的效果较好,但dSPE法的平均回收率远低于Sin-QuEChERS Nano法,而且前处理操作中dSPE法比Sin-QuEChERS Nano法多了一步纯化管净化步骤,单个样品前处理时间需多耗时10 min,不适于批量样品的“萃取净化一体式”检测。其次,dSPE中的萃取管内含有高含量石墨化炭黑,对平面结构农药有一定的吸附^[30]^,导致菊酯类目标农药的回收率偏低,而Sin-QuEChERS Nano净化柱中PSA等固相吸附材料与MWCNTs有效聚合,比石墨化炭黑具有更强的吸附和净化能力^[31]^。同时,Sin-QuEChERS Nano法加入的缓冲盐萃取包也能有效控制有机相的酸碱度,改善对酸敏感农药的回收率,提取效果也优于普通盐包。

另外本研究分别比较了3款市售Sin-QuEChERS Nano商品化柱,分别是简单基质净化柱、复杂基质净化柱及中药净化柱,从净化效果及回收率结果来看,只有中药净化柱(Sin-QuEChERS Nano柱)可以满足石斛类基质的净化。其次,为了突出本净化方法的适用范围广,研究中分别选取不同部位(茎、叶、花)的金钗石斛样品,经提取后过Sin-QuEChERS Nano柱净化,观察其净化效果,具体如图4所示。

**图4:**
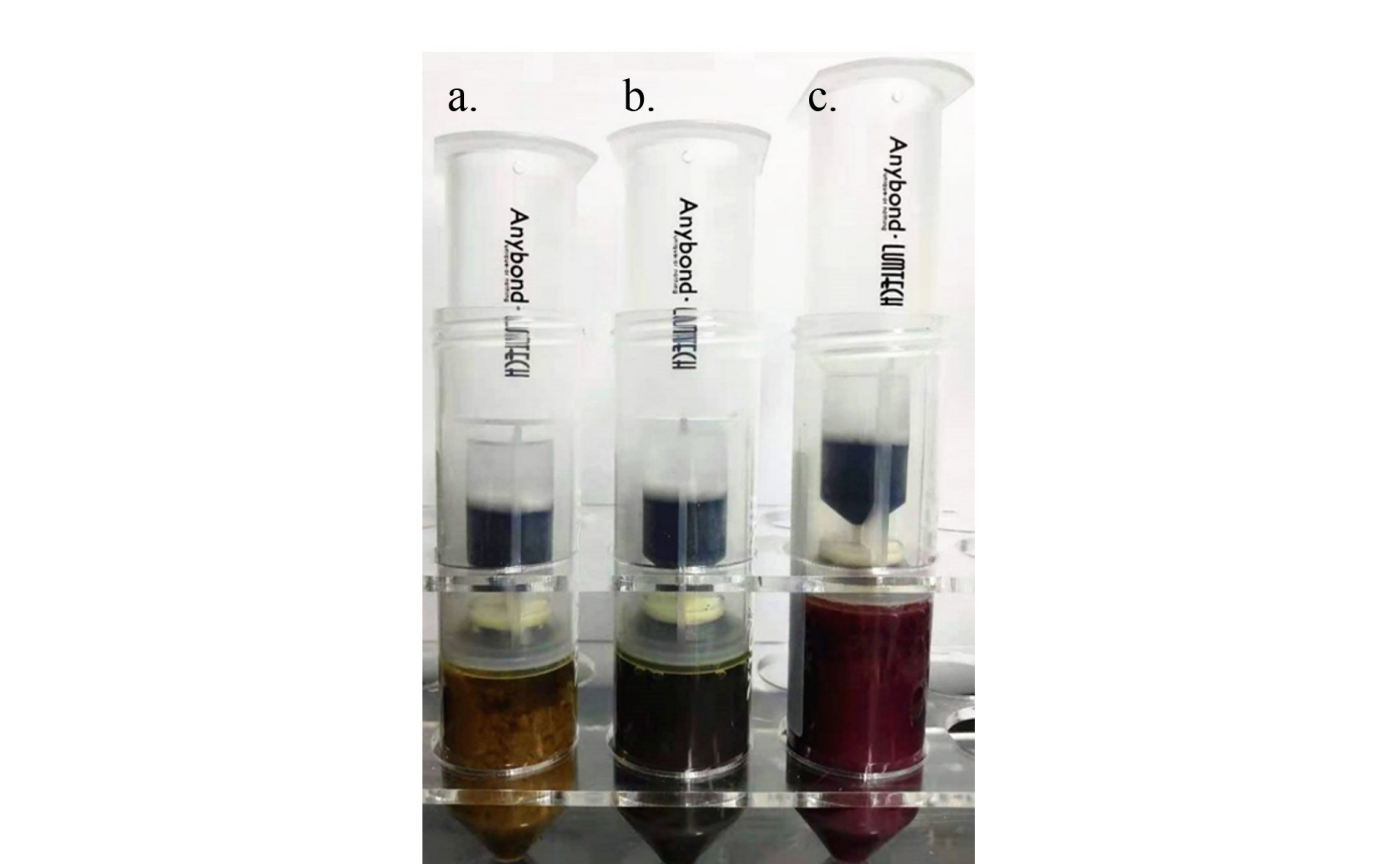
采用Sin-QuEChERS Nano柱净化后石斛样品的净化效果图

从图4可以看出,Sin-QuEChERS Nano柱储液管内净化液均为澄清透明液体,对金钗石斛的茎、叶、花净化效果都较为显著,所以后续实验将Sin-QuEChERS Nano柱作为首选石斛样品净化柱。

### 2.2 提取方式及提取溶剂的选择

Sin-QuEChERS Nano法是在QuEChERS法基础上改良出的一种全新的样品前处理方法,常应用于含水量较大的蔬菜和水果,当基质的含水量小于20%时,该法会影响细胞间的通透性,不利于农药组分的析出。但是加入水量过多会导致后续液液分层不明显,加入水量过少又达不到改变通透性的效果,在本研究中只有加入5 mL水浸泡20 min后才能全部浸没不同性状及部位的石斛样品(粉状、叶、花、鲜茎等)。其次,本研究石斛样品前处理过程中以加5 mL水及未加水两种操作方式下的平均回收率作为考察指标,进一步考察了目标组分的提取效率。结果显示:样品加入5 mL水浸泡20 min再进行后续操作后,19种水溶性农药回收率有所提高,其回收率范围由62.6%~107.6%提高至68.7%~115.5%,其平均回收率提高了11.3%。所以本研究称量样品后需加入5 mL水浸泡20 min后再进行后续的前处理操作。

此外,提取农药的有机溶剂选择性很多,根据目标组分极性的不同及基质的影响使用较多的试剂主要有1%乙酸乙腈、丙酮等,本研究考察Sin-QuEChERS Nano法中这两种不同提取溶剂的提取效果,结合提取液颜色及提取率作为考察指标。结果如图5所示:当用1%乙酸乙腈、丙酮作为提取溶剂时,各目标组分的提取率均大于68.7%,但用丙酮作为提取溶剂时,提取液的颜色较深且体积较少,不利于后续的净化操作,所以后续的实验操作均以1%乙酸乙腈作为提取溶剂。

**图5:**
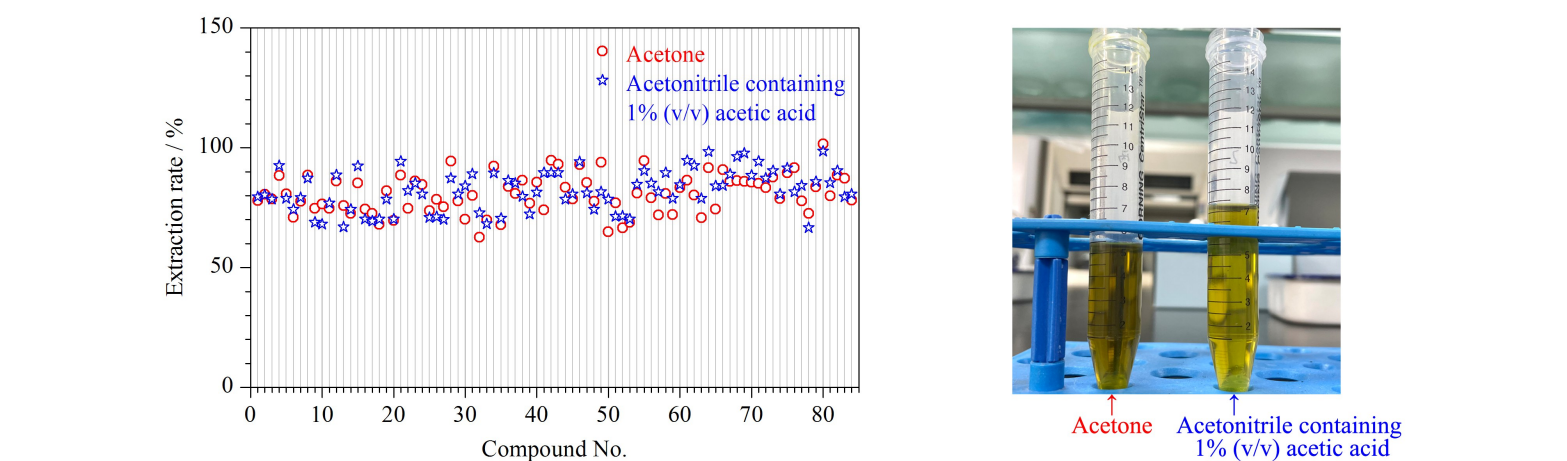
不同提取溶剂对提取率的影响及提取液颜色的比较

### 2.3 基质效应

本研究为考察84种农药的基质效应,分别采用净化效果较好的dSPE法和Sin-QuEChERS Nano法进行前处理操作,石斛中目标农药的基质效果如图6所示。结果显示:Sin-QuEChERS Nano法的ME为101%~158%,其中40种农药的ME>120%,而dSPE法的ME范围为109%~189%,其中54种农药的ME>120%。由此可见,与液相色谱-质谱法不同的是,气相色谱-串联质谱法的基质效应大多数以增强为主,Sin-QuEChERS Nano法比dSPE法更有效减小了基质效应。所以本研究为了减少基质效应对定量的影响而采用基质匹配工作曲线外标法定量^[32]^。

**图6:**
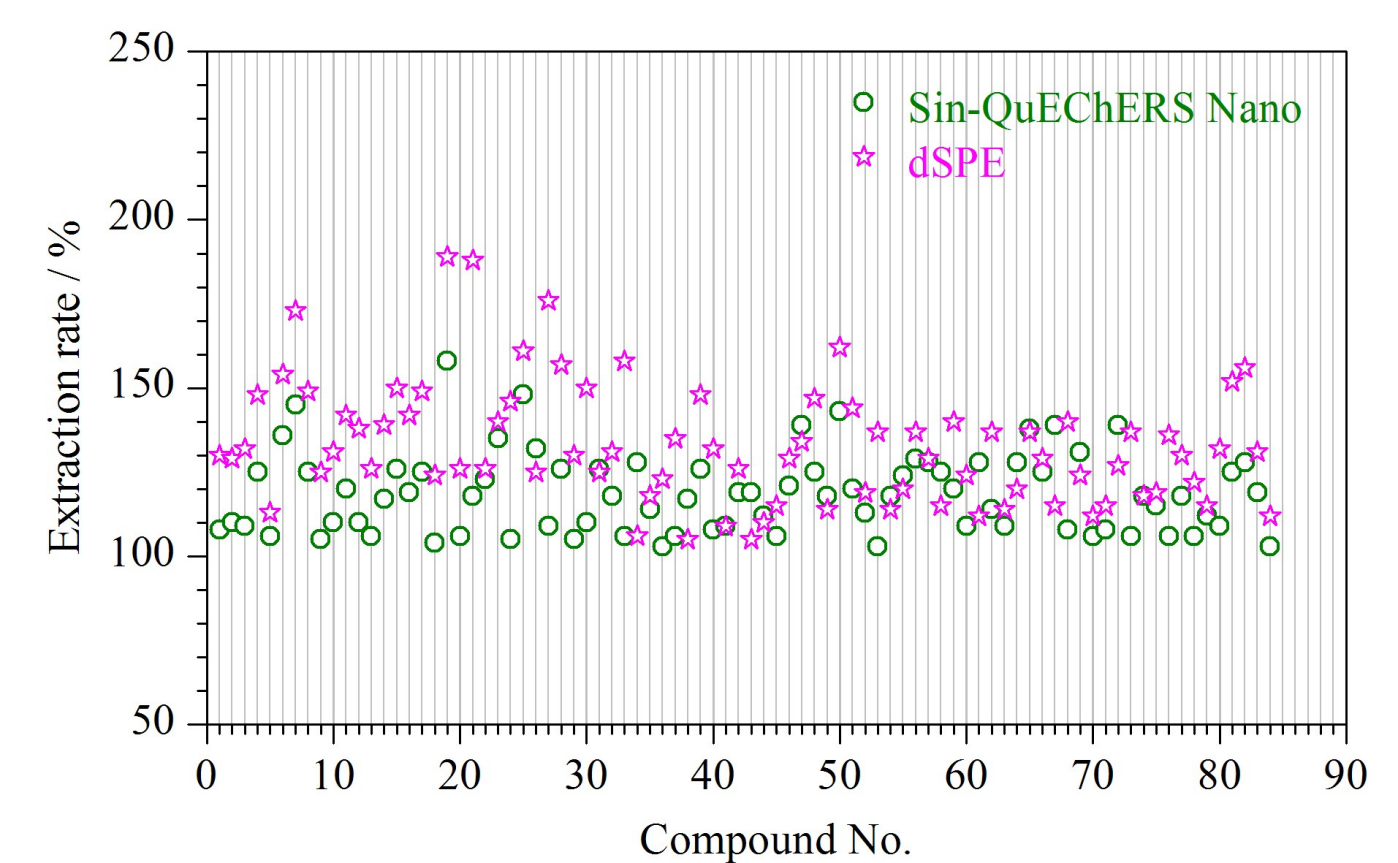
使用不同净化方法时石斛中目标农药的基质效应

### 2.4 线性范围、检出限、定量限及加标回收率

在GC-MS/MS确证的实验条件下,取石斛空白样品按样品制备处理后得到空白基质,分别加入6个不同浓度的混合标准溶液,在上述的质谱条件下进行测定。以目标农药的峰面积*Y*为纵坐标,相对应的质量浓度*X*(μg/L)为横坐标绘制标准曲线。同时,采用空白石斛样品中添加目标物的方法,以3倍和10倍信噪比(*S/N*)对应的含量确定检出限(LOD)和定量限(LOQ)。取数份经GC-MS/MS验证无目标物的石斛空白样品,分别添加50 μL和250 μL不同质量浓度的混合标准使用液,按照上述优化后的方法进行样品前处理,并上机分析,平行测定6次。

84种化合物的线性方程、线性范围、相关系数(*r*^2^ )、检出限、定量限、加标回收率及相对标准偏差如表2所示。84种目标物线性关系良好(*r*^2^ >0.99)。采用加标回收的方法确定方法的检出限及定量限分别为1.5~5.8 μg/kg和5.0~15.0 μg/kg; 84种化合物加标回收率为68.7%~116.2%, RSD(*n*=6)为5.2%~14.8%。表明本方法重复性良好,可满足实际检测的需要。

**表2:** 84种化合物的线性方程、线性范围、相关系数、检出限、定量限、加标回收率及相对标准偏差(*n*=6)

Pesticide	Regression equation		Linear range/ (μg/L)		*r*^2^		LOD/ (μg/ kg)		LOQ/ (μg/ kg)		50 μL					250 μL						
											Recovery/ %		RSD/ %			Recovery/ %					RSD/ %	
Methomyl	*Y*=3.67×10^4^*X*-1.41×10^3^		105.5-3375.0		0.9955		3.0		10.0		94.5		7.6			100.7					13.9	
Metolcarb	*Y*=4.82×10^4^*X*-3.5×10^3^		12.5-400.0		0.9981		1.5		5.0		85.6		10.4			74.5					8.9	
Entrofolan	*Y*=4.77×10^5^*X*-9.0×10^3^		12.5-400.0		0.9991		1.5		5.0		78.8		7.3			90.4					9.6	
Metrifonate	*Y*=3.06×10^5^*X*-2.07×10^3^		16.3-520.8		0.9997		1.5		5.0		87.6		7.6			78.9					8.9	
Dichlorvos	*Y*=1.07×10^6^*X*-6.46×10^4^		15.6-500.0		0.9982		1.5		5.0		93.9		5.8			78.7					7.4	
Carbofuran	*Y*=1.11×10^6^*X*-2.36×10^4^		25.0-800.0		0.9968		1.5		5.0		95.7		14.8			81.1					12.4	
Biphenyl	*Y*=1.81×10^7^*X*-4.99×10^5^		6.3-200.0		0.9952		3.0		10.0		75.1		7.4			113.6					6.2	
Methamidophos	*Y*=1.46×10^6^*X*-1.80×10^4^		43.8-1400.0		0.9979		1.5		5.0		79.1		8.1			88.6					8.1	
Demeton	*Y*=5.89×10^5^*X*-1.49×10^4^		6.3-200.0		0.9978		3.0		10.0		100.7		6.5			87.9					8.3	
Ethoprophos	*Y*=6.19×10^5^*X*-4.90×10^4^		6.3-200.0		0.9974		1.5		5.0		75.1		6.4			105.2					11.7	
Carbaryl	*Y*=6.77×10^5^*X*-9.21×10^4^		46.9-1500.0		0.9931		5.8		20.0		103.2		7.3			76.6					6.5	
Fenobucarb	*Y*=5.28×10^6^*X*-1.91×10^5^		12.5-400.0		0.9983		3.0		10.0		108.8		13.5			78.8					7.6	
Chlordimeform	*Y*=3.54×10^5^*X*-2.77×10^4^		6.3-200.0		0.9983		1.5		5.0		88.4		6.2			88.6					6.3	
Phorate	*Y*=2.03×10^6^*X*-2.47×10^4^		21.9-700.0		0.9958		1.5		5.0		79.4		6.6			102.4					9.4	
Propoxur	*Y*=2.14×10^6^*X*-1.31×10^5^		12.5-400.0		0.9916		1.5		5.0		68.7		5.4			84.4					6.2	
Sulfotep	*Y*=3.22×10^5^*X*-6.92×10^3^		6.3-200.0		0.9978		3.0		10.0		82.3		13.8			116.2					13.9	
*α*-Hexachlorocyclohexane	*Y*=1.52×10^6^*X*-1.71×10^4^		15.6-500.0		0.9948		3.0		10.0		78.7		7.2			90.3					9.2	
Bendiocarb	*Y*=1.73×10^5^*X*-7.87×10^4^		18.8-600.0		0.9982		3.0		10.0		106.1		10.9			73.2					7.8	
Quintozine	*Y*=1.41×10^6^*X*-4.70×10^4^		31.3-1000.0		0.9983		4.4		15.0		92.2		10.6			81.1					10.5	
Terbufos	*Y*=1.97×10^5^*X*-4.35×10^3^		6.3-200.0		0.9998		3.0		10.0		113.2		10.2			99.7					11.1	
Dithianon	*Y*=1.21×10^5^*X*-2.08×10^4^		15.6-500.0		0.9987		1.5		5.0		74.3		13.8			86.1					6.2	
Lindane	*Y*=1.23×10^6^*X*-4.62×10^3^		15.6-500.0		0.9961		1.5		5.0		73.8		12.1			87.5					5.4	
Omethoate	*Y*=1.82×10^4^*X*-1.46×10^3^		15.6-500.0		0.9952		3.0		10.0		83.2		11.2			78.3					10.9	
Atrazine	*Y*=5.12×10^5^*X*-2.62×10^3^		16.3-520.8		0.9915		3.0		10.0		97.4		13.2			69.8					13.2	
Cyromazine	*Y*=1.11×10^5^*X*-1.27×10^4^		16.3-520.8		0.9946		3.0		10.0		86.3		8.9			99.2					13.9	
Pirimicarb	*Y*=2.52×10^6^*X*-4.84×10^4^		15.6-500.0		0.9941		3.0		10.0		85.1		8.9			105.4					9.2	
Isazofos	*Y*=8.21×10^5^*X*-1.09×10^4^		31.3-1000.0		0.9945		1.5		5.0		104.5		12.3			85.8					10.5	
Aldrin	*Y*=6.57×10^5^*X*+2.79×10^3^		15.6-500.0		0.9974		1.5		5.0		115.0		8.1			95.1					7.7	
Chlorpyrifos-methyl	*Y*=1.76×10^6^*X*-7.13×10^4^		31.3-1000.0		0.9988		1.5		5.0		88.4		7.6			76.2					13.2	
Dimethoate	*Y*=4.51×10^6^*X*-1.60×10^5^		12.5-400.0		0.9986		1.5		5.0		78.6		9.8			101.4					13.2	
Monocrotophos	*Y*=7.50×10^4^*X*-3.36×10^3^		9.4-300.0		0.9932		3.0		10.0		86.8		7.3			78.4					5.2	
Fenchlorphos	*Y*=2.03×10^6^*X*-6.39×10^4^		16.3-520.8		0.9992		3.0		10.0		76.8		6.7			79.4					6.1	
Indoxacarb	*Y*=8.55×10^3^*X*-1.27×10^3^		16.3-520.8		0.9964		3.0		10.0		109.5		12.3			97.1					7.6	
Pirimiphos-methyl	*Y*=1.46×10^6^*X*-3.13×10^4^		16.3-520.8		0.9997		3.0		10.0		68.1		11.4			84.9					6.9	
Chlorothalonil	*Y*=2.55×10^5^*X*-6.41×10^4^		31.3-1000.0		0.9998		3.0		10.0		74.8		7.6			95.6					5.4	
*β*-Hexachlorocyclohexane	*Y*=2.80×10^6^*X*-2.95×10^4^		15.6-500.0		0.9982		3.0		10.0		83.3		8.4			100.7					7.2	
Clorpyrifos	*Y*=3.51×10^6^*X*-9.51×10^4^		31.3-1000.0		0.9992		3.0		10.0		88.9		9.3			89.5					6.9	
Parathion-methyl	*Y*=1.22×10^6^*X*-4.39×10^4^		21.9-700.0		0.9932		3.0		10.0		87.3		7.7			88.3					7.6	
Fenthion	*Y*=4.94×10^6^*X*-1.63×10^5^		31.3-1000.0		0.9976		3.0		10.0		79.5		8.7			98.2					7.6	
Dicofol	*Y*=2.23×10^6^*X*-1.74×10^5^		15.6-500.0		0.9952		1.5		5.0		96.9		8.3			115.4					13.8	
*δ*-Hexachlorocyclohexane	*Y*=2.15×10^6^*X*-3.18×10^4^		15.6-500.0		0.9997		1.5		5.0		80.6		7.3			93.2					8.2	
Malaoxon	*Y*=2.42×10^6^*X*-5.03×10^4^		15.6-500.0		0.9921		1.5		5.0		88.3		9.9			86.1					10.9	
Pesticide	Regression equation		Linear range/ (μg/L)		*r* ^2^		LOD/ (μg/ kg)		LOQ/ (μg/ kg)		50 μL					250 μL						
											Recovery/ %		RSD/ %			Recovery/ %					RSD/ %	
Fenitrothion	*Y*=9.94×10^5^*X*-5.30×10^4^		31.3-1000.0		0.9933		3.0		10.0		86.3		6.3			89.7					8.3	
Parathion	*Y*=1.13×10^6^*X*-1.60×10^4^		21.9-700.0		0.9977		1.5		5.0		80.3		10.3			80.7					7.2	
*a*-Endosulfan	*Y*=4.75×10^5^*X*-1.51×10^4^		15.6-500.0		0.9941		1.5		5.0		84.7		9.2			82.9					10.9	
Isofenphos-methyl	*Y*=5.27×10^5^*X*-1.88×10^4^		6.3-200.0		0.9998		3.0		10.0		86.1		12.9			103.6					7.5	
Phorat-sulfoxide	*Y*=2.42×10^5^*X*-9.94×10^3^		16.3-520.8		0.9971		3.0		10.0		105.8		11.9			86.5					8.9	
Quintiofos	*Y*=1.14×10^6^*X*-2.05×10^4^		15.6-500.0		0.9963		1.5		5.0		97.2		13.7			70.5					4.9	
Isocarbophos	*Y*=3.83×10^6^*X*-1.04×10^5^		31.3-1000.0		0.9964		1.5		5.0		109.4		6.8			97.4					14.7	
*p*,*p'*-Dichlorodiphenyldichloroethane	*Y*=2.36×10^6^*X*-5.91×10^3^		15.6-500.0		0.999		3.0		10.0		84.7		5.6			96.1					6.2	
Phorate-sulfone	*Y*=3.78×10^6^*X*-8.48×10^4^		16.3-520.8		0.9981		3.0		10.0		95.1		15.2			111.7					8.3	
Dieldrin	*Y*=2.91×10^5^*X*-1.74×10^3^		15.6-500.0		0.9992		3.0		10.0		78.3		11.3			74.2					10.4	
Profenofos	*Y*=8.37×10^5^*X*-4.59×10^4^		31.3-1000.0		0.9991		3.0		10.0		82.1		7.2			82.9					6.5	
Procymidone	*Y*=6.95×10^6^*X*-1.02×10^5^		31.3-1000.0		0.9985		3.0		10.0		79.9		8.2			100.6					5.2	
Methidathion	*Y*=5.88×10^6^*X*-2.21×10^5^		31.3-1000.0		0.9922		3.0		10.0		78.4		7.8			103.3					13.9	
Endrin-ketone	*Y*=1.43×10^5^*X*-1.42×10^4^		16.3-520.8		0.9972		3.0		10.0		94.9		6.1			87.1					12.8	
Phosfolan-methyl	*Y*=2.35×10^5^*X*-2.84×10^4^		9.4-300.0		0.9928		3.0		10.0		90.7		10.1			74.5					6.3	
*o*,*p'*-Dichlorodiphenyltrichloroethane	*Y*=2.55×10^6^*X*-4.74×10^4^		15.6-500.0		0.9964		3.0		10.0		80.3		10.4			79.8					11.9	
Fipronil-desulfinyl	*Y*=6.21×10^5^*X*-1.23×10^3^		6.3-200.0		0.9988		3.0		10.0		76.8		11.3			112.5					11.7	
Fenamiphos	*Y*=2.65×10^6^*X*-1.28×10^3^		6.3-200.0		0.9918		3.0		10.0		70.9		9.9			73.2					8.5
Nitrofen	*Y*=5.75×10^5^*X*-5.21×10^3^		15.6-500.0		0.9940		3.0		10.0		87.4		5.8			115.1					7.2	
*p*,*p'*-Dichlorodiphenyldichloroethane	*Y*=5.66×10^6^*X*-8.58×10^4^		15.6-500.0		0.9999		3.0		10.0		94.2		12.7			91.1					9.5	
*β*-Endosulfan	*Y*=3.72×10^5^*X*-7.73×10^3^		15.6-500.0		0.9983		3.0		10.0		97.5		9.5			109.8					6.7	
Ethion	*Y*=2.00×10^6^*X*-1.12×10^4^		16.3-520.8		0.9965		1.5		5.0		81.7		12.9			97.8					13.2	
*p*,*p'*-Dichlorodiphenyltrichloroethane	*Y*=1.47×10^6^*X*-4.22×10^4^		15.6-500.0		0.9922		3.0		10.0		71.8		7.5			90.4					9.9	
*τ*-Fluvalinate	*Y*=2.81×10^4^*X*-21.5		31.3-1000.0		0.9979		3.0		10.0		91.1		9.1			112.4					5.4	
Chlorfenapyr	*Y*=5.92×10^5^*X*-1.37×10^4^		16.3-520.8		0.9998		3.0		10.0		70.2		14.4			108.2					9.6	
Fipronil-sulfide	*Y*=1.10×10^5^*X*-6.22×10^3^		6.3-200.0		0.9914		3.0		10.0		78.6		11.2			69.5					14.1	
Fipronil	*Y*=1.06×10^6^*X*-3.72×10^4^		6.3-200.0		0.9949		3.0		10.0		100.8		12.9			89.3					6.5	
Bifenthrin	*Y*=2.51×10^6^*X*-2.05×10^5^		31.3-1000.0		0.9986		3.0		10.0		109.8		6.3			72.2					6.5	
Triazophos	*Y*=2.03×10^6^*X*-9.87×10^4^		31.3-1000.0		0.9977		3.0		10.0		99.4		6.7			88.1					7.6	
Thiodan-Sulfate	*Y*=8.97×10^5^*X*-1.72×10^4^		15.6-500.0		0.9981		3.0		10.0		103.2		7.9			80.3					14.8	
Fenpropathrin	*Y*=1.17×10^6^*X*-1.50×10^4^		31.3-1000.0		0.9983		3.0		10.0		74.1		10.5			83.4					9.2	
Phosphonothioic acid	*Y*=3.14×10^6^*X*-3.66×10^4^		31.3-1000.0		0.9997		3.0		10.0		108.9		7.9			92.5					7.8	
Phosmet	*Y*=5.42×10^5^*X*-3.39×10^4^		31.3-1000.0		0.9949		3.0		10.0		109.2		9.1			99.5					9.6	
Cyhalothrin	*Y*=1.85×10^6^*X*+2.07×10^4^		31.3-1000.0		0.9987		4.4		15.0		82.1		5.4			93.4					9.4	
Permethrin	*Y*=3.55×10^5^*X*+7.59×10^2^		31.3-1000.0		0.9989		3.0		10.0		93.1		12.8			82.4					11.2	
Phosalone	*Y*=6.84×10^5^*X*-1.58×10^4^		31.3-1000.0		0.9925		1.5		5.0		96.1		14.1			72.5					8.9	
Fipronil-sulfone	*Y*=5.16×10^5^*X*+8.45×10^3^		6.3-200.0		0.9927		3.0		10.0		86.8		6.2			83.2					10.2	
Cyfluthrin	*Y*=2.75×10^6^*X*-4.94×10^2^		31.3-1000.0		0.9953		4.4		15.0		92.7		12.0			81.8					8.3	
Cypermethrin	*Y*=7.86×10^6^*X*+1.86×10^3^		31.3-1000.0		0.9942		3.0		10.0		80.6		12.3			106.5					7.4	
Coumaphos	*Y*=1.21×10^5^*X*-9.75×10^3^		15.6-500.0		0.9974		1.5		5.0		106.7		6.6			93.8					10.7	
Fenvalerate	*Y*=1.39×10^6^*X*-2.21×10^4^		31.3-1000.0		0.9935		3.0		10.0		84.5		7.9			91.3					11.3	
Deltamethrin	*Y*=6.53×10^3^*X*+1.87×10^2^		31.3-1000.0		0.9978		3.0		10.0		96.8		6.3			108.7					6.1	

*Y*: peak area; *X*: mass concentration, μg/L.

### 2.5 实际样品测试

采用本研究建立的方法对贵州省安龙县、荔波县、独山县、沿河县采集的共80份样品进行监测,样品类别包含石斛枫斗、金钗石斛(花、茎、叶)及铁皮石斛(花、茎、叶、粉、片)。

结果表明,其中12份样品中检出农药残留,检出率为15%,检出的农药频次较高的有毒死蜱(0.08~0.5 mg/kg)、百菌清(0.06~3.2 mg/kg)、腐霉利(0.03~0.15 mg/kg)、甲基对硫磷(0.04~0.23 mg/kg)、氯氟氰菊酯(0.10~2.68 mg/kg)、克百威(0.015~0.02 mg/kg),同时检出农药的残留量均未超过国家标准GB 2763-2021《食品安全国家标准食品中农药最大残留限量》及地方标准DBS 52/048-2020《食品安全地方标准铁皮石斛茎》规定的最大残留限量值。图7为阳性样品中典型目标物的总离子流图。

**图7:**
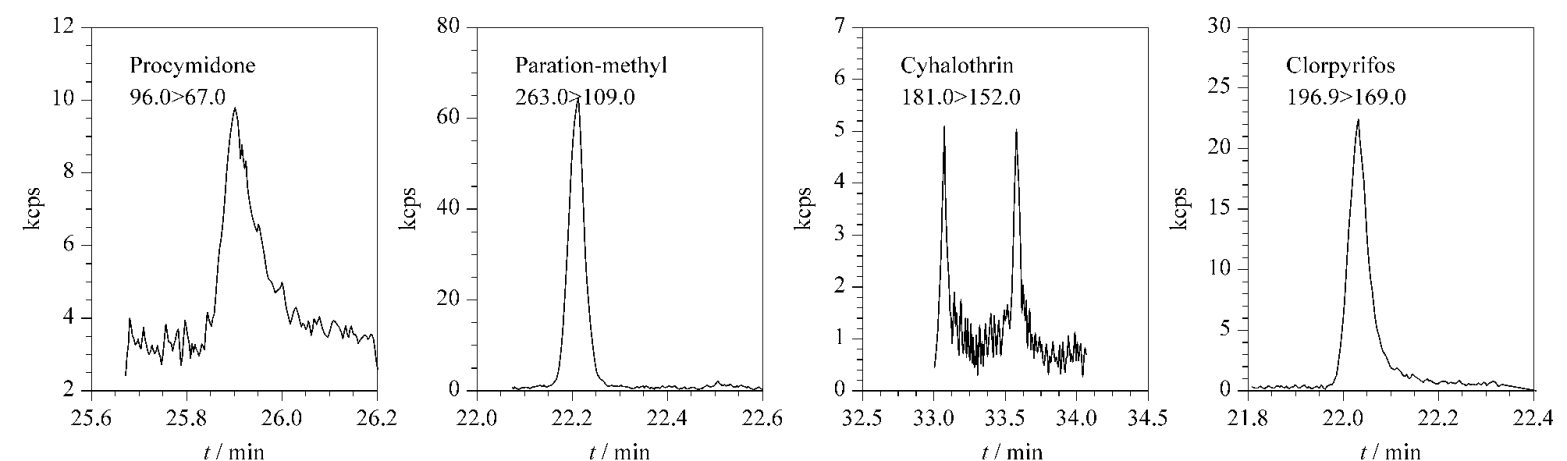
阳性样品中典型目标物的总离子流图

## 3 结论

本研究基于Sin-QuEChERS Nano柱开发了一种快速筛查石斛中多种农药残留的方法。通过对Sin-QuEChERS Nano与经典dSPE、SPE、QuEChERS法的净化效果、提取回收率进行比较及方法学参数验证,表明Sin-QuEChERS Nano柱在净化效果方面表现更好。该法充分简化了样品的净化过程,同时提高了检测效率和准确性,更加适合批量样品中多组分农药残留的筛查及确证工作。

## References

[1] Karoojee S, Noypitak S, Abdullakasim S. Hortic Environ Biotechnol, 2020, 11(6): 1

[2] Kolomeitseva G L, Babosha A V, Ryabchenko A S, et al. Protoplasma, 2020, 258(2): 301 33070242 10.1007/s00709-020-01573-2

[3] Shao S C, Wang Q X, Beng K C, et al. Mycorrhiza, 2020, 30(9): 529 32562087 10.1007/s00572-020-00964-w

[4] Xu Z L, Li L X Y, Xu Y, et al. Food Chem, 2021, 343: 128490 33158673 10.1016/j.foodchem.2020.128490

[5] Liu C Z, Chen W, Wang M X, et al. Chin J Nat Med, 2020, 18(6): 446 32503736 10.1016/S1875-5364(20)30052-2

[6] Li M X, Yue H, Wang Y Q, et al. Int J Biol Macromol, 2020, 149: 717 32014483 10.1016/j.ijbiomac.2020.01.305

[7] Liang J, Li H L, Chen J Q. Pharmacol Res, 2019, 148(8): 104417 31473343 10.1016/j.phrs.2019.104417

[8] Zheng S G, Hu Y D, Zhao R X, et al. J Chromatogr B, 2020, 1140: 122017 10.1016/j.jchromb.2020.122017 32050157

[9] Wang C X, Xu L, Guo X X, et al. J Food Process Preserv, 2018, 42(5): 1

[10] Ding G, Xu G H, Zhang W C, et al. Eur Food Res Technol, 2008, 227(4): 1283

[11] Guarda P M, Pontes A M S, Domiciano R D S, et al. Arch Environ Contam Toxicol, 2020, 79(4): 524 33150460 10.1007/s00244-020-00770-7

[12] Zhan X P, Ma L, Huang L Q, et al. J Chromatogr B, 2017, 1060: 281

[13] Lopez S H, Dias J, De K A. Food Control, 2020, 115: 107289

[14] Wang S C, Qi P P, Di S S, et al. Anal Chim Acta, 2019, 1074: 108 31159930 10.1016/j.aca.2019.04.063

[15] Bandforuzi S R, Hadjmohammadi M R. Anal Chim Acta, 2019, 1078: 90 31358233 10.1016/j.aca.2019.06.026

[16] Li L, Wang X H, Sun Y, et al. Talanta, 2018, 179: 512 29310269 10.1016/j.talanta.2017.11.017

[17] Park J I, Al-Mutairi A, Marafie A M J, et al. J Ind Eng Chem, 2016, 34: 204

[18] Guo J G, Tong M M, Tang J, et al. Food Chem, 2019, 274: 452 30372964 10.1016/j.foodchem.2018.08.134

[19] Mogaddam M R A, Farajzadeh M A, Damirchi S A, et al. J Chromatogr A, 2020, 1630: 461523 32920246 10.1016/j.chroma.2020.461523

[20] Villaverde J J, Sevilla-Morán B, López-Goti C, et al. Molecules, 2018, 23: 1 10.3390/molecules23082009 PMC6222645 30103524

[21] Tuzimski T, Szubartowski S. Molecules, 2019, 24(11): 2093 10.3390/molecules24112093 PMC6600471 31159388

[22] Sharmili K, Jinap S, Sukor R. Food Control, 2016, 70: 152

[23] Kim Y A, Abd El-Aty A M, Rahman M M, et al. J Chromatogr B, 2018, 1076: 130 10.1016/j.jchromb.2018.01.015 29406026

[24] Chen J N, Lian Y J, Zhou Y R, et al. Molecules, 2019, 24: 2918

[25] Zhu B Q, Xu X Y, Luo J W, et al. Food Chem, 2019, 276: 202 30409585 10.1016/j.foodchem.2018.09.152

[26] Zou N, Han Y T, Li Y J, et al. J Agric Food Chem, 2016, 64(31): 6061 26651870 10.1021/acs.jafc.5b05132

[27] Li Y J, An Q S, Zhang C P, et al. Molecules, 2020, 25(15): 3391

[28] Yang J R, Chen H H, Nie Q X, et al. Int J Biol Macromol, 2020, 164: 1939 32763406 10.1016/j.ijbiomac.2020.08.007

[29] Kecojevic I, Dekic S, Lazovic M, et al. Food Control, 2021, 123: 1

[30] Kinross A D, Hageman K J, Doucette W J, et al. J Chromatogr A, 2020, 1627: 461414 32823112 10.1016/j.chroma.2020.461414

[31] Song L, Han Y T, Yang J, et al. Food Chem, 2019, 279: 237 30611486 10.1016/j.foodchem.2018.12.017

[32] Gong G P, Dang T T, Deng Y N, et al. Int. J. Biol. Macromol, 2018, 109: 611 29222018 10.1016/j.ijbiomac.2017.12.017

